# Selenoproteome Expression Studied by Non-Radioactive Isotopic Selenium-Labeling in Human Cell Lines

**DOI:** 10.3390/ijms22147308

**Published:** 2021-07-07

**Authors:** Jordan Sonet, Anne-Laure Bulteau, Zahia Touat-Hamici, Maurine Mosca, Katarzyna Bierla, Sandra Mounicou, Ryszard Lobinski, Laurent Chavatte

**Affiliations:** 1 Institut des Sciences Analytiques et de Physico-Chimie Pour l’Environnement et les Matériaux (IPREM), Universite de Pau, CNRS, E2S, UMR 5254, Hélioparc, 64053 Pau, France; jordan.sonet@gmail.com (J.S.); mosca.maurine64@gmail.com (M.M.); katarzyna.bierla@univ-pau.fr (K.B.); sandra.mounicou@univ-pau.fr (S.M.); ryszard.lobinski@univ-pau.fr (R.L.); 2 LVMH Recherche, Life Science Department, 185 Avenue de Verdun, 45800 Saint Jean de Braye, France; abulteau@research.lvmh-pc.com; 3 Centre de Génétique Moléculaire, CGM, CNRS, UPR3404, 91198 Gif-sur-Yvette, France; ztouat@gmail.com; 4 Laboratory of Molecular Dietetics, I.M. Sechenov First Moscow State Medical University, 19945 Moscow, Russia; 5 Chair of Analytical Chemistry, Warsaw University of Technology, Noakowskiego 3, 00-664 Warsaw, Poland; 6 Centre International de Recherche en Infectiologie (CIRI), 69007 Lyon, France; 7 Institut National de la Santé et de la Recherche Médicale (INSERM), Unité U1111, 69007 Lyon, France; 8 Ecole Normale Supérieure de Lyon, 69007 Lyon, France; 9 Université Claude Bernard Lyon 1 (UCBL1), 69622 Lyon, France; 10 Centre National de la Recherche Scientifique (CNRS), Unité Mixte de Recherche 5308 (UMR5308), 69007 Lyon, France

**Keywords:** selenoproteome, selenoprotein hierarchy, nonradioactive isotopes, SEC-ICP MS, glutathione peroxidase, thioredoxin reductase, SECIS, translation regulation

## Abstract

Selenoproteins, in which the selenium atom is present in the rare amino acid selenocysteine, are vital components of cell homeostasis, antioxidant defense, and cell signaling in mammals. The expression of the selenoproteome, composed of 25 selenoprotein genes, is strongly controlled by the selenium status of the body, which is a corollary of selenium availability in the food diet. Here, we present an alternative strategy for the use of the radioactive ^75^Se isotope in order to characterize the selenoproteome regulation based on (i) the selective labeling of the cellular selenocompounds with non-radioactive selenium isotopes (^76^Se, ^77^Se) and (ii) the detection of the isotopic enrichment of the selenoproteins using size-exclusion chromatography followed by inductively coupled plasma mass spectrometry detection. The reliability of our strategy is further confirmed by western blots with distinct selenoprotein-specific antibodies. Using our strategy, we characterized the hierarchy of the selenoproteome regulation in dose–response and kinetic experiments.

## 1. Introduction

The vital role of the essential trace element selenium (Se) in human health has been widely reported. Selenium deficiency is often evoked in the context of cancer, cardiac function, muscular disorders, infections, neurodegenerative disease, and aging [1,2,3,4,5,6,7,8]. Despite the essential nature of selenium, the range of its optimal intake is quite narrow and its toxicity can be quite easily achieved depending on its chemical speciation. Selenium is co-translationally incorporated into a group of proteins, referred to as selenoproteins [9], in the form of a rare amino-acid, selenocysteine (Sec), using a UGA codon, normally used as a stop signal for protein synthesis [10,11]. Twenty-five selenoprotein genes have been identified in the human genome and give rise to selenoproteome expression in tissue and cell-line specific patterns [12]. The selenoproteome is primarily regulated by the bioavailability of selenium from food or from a cell culture medium. This selenium-dependent regulation follows a specific hierarchy that stipulates that “house-keeping” members are kept constant at the expense of “stress-regulated” members, which respond to changes in the selenium level [6,8,13]. Other stimuli, including H_2_O_2_-induced oxidative stress and replicative senescence, are also able to modulate selenoprotein expression but follow distinct hierarchies [4,14,15,16,17,18].

Selenoprotein biosynthesis follows a non-conventional mechanism which involves the recoding of UGA, normally used as a termination signal, in a Sec insertion codon. To do so, the translation machinery has evolved a sophisticated strategy to cope with using UGA as Sec for selenoprotein mRNAs, while maintaining its use as a stop codon for all other cellular mRNAs. This non-canonical pathway essentially relies on two RNA molecules, namely: (i) the Sec insertion sequence (SECIS) located in the 3′ untranslated region (UTR) of selenoprotein mRNAs; (ii) the Sec-tRNA^[Ser]Sec^; and (iii) their interacting partners (as reviewed in [10,11,13,19,20,21]). Only one Sec residue is present in each selenoprotein (except for SELENOP) [12]. Well-characterized members are glutathione peroxidases (GPX), thioredoxin reductases (TXNRD), iodothyronine deiodinases (DIO), methionine sulfoxide reductase (MSR), and endoplasmic reticulum (ER) selenoproteins (SELENOF, SELENOS, SELENOK, SELENON, SELENOM, and SELENOT). Additionally, selenophosphate synthetase 2 (SEPHS2) is implicated in the synthesis of the selenocysteine precursor, selenophosphate (SePO_3_^3−^), and therefore participates in the selenoprotein biosynthesis pathway. The function of about one-third of all selenoproteins remains largely uncharacterized. Human embryonic kidney 293 (HEK293) cells express a wide range of selenoproteins that are highly responsive to selenium levels [15,22]. This cell line offers a validated model for the prioritized selenium-dependent regulation of selenoproteins. Growth media with selenium limiting conditions (3 nM) were engineered and validated [15,16,23]. In particular, the response to selenium levels was reported for the members of the GPX family. It is referred to as the selenoprotein hierarchy and has yet to be characterized for the entire selenoproteome [10].

Selenoprotein detection has long been limited to western immunoblots and radioactivity. Recently, the use of inductively coupled plasma-mass spectrometry (ICP MS) coupled to protein separation methods has proved efficient for the simultaneous detection of abundant selenoproteins [24,25,26,27,28]. First, western immunoblots are limited to the availability of antibodies. Although highly specific and semi-quantitative, this method does not provide information about the relative abundance between selenoproteins. Alternatively, radioactive ^75^Se (in the form of selenite) has been widely used to label selenoproteins in vivo and in vitro [29], and provides relative abundance data for abundant selenoproteins. Radiolabeled proteins are separated by different biochemical methods, including chromatography or SDS-PAGE, and are revealed by gamma-counting or autoradiography. ^75^Se is a gamma-emitter with a rather long half-life (120 days) and its use is restricted to a few laboratories. In addition, only abundant selenoproteins are visible by the autoradiography of SDS-PAGE. The presence of selenium in a molecule can be otherwise detected by elemental ion mass spectrometry, such as ICP MS, which is able to differentiate and quantify all natural isotopes of selenium. Indeed, selenium has six stable isotopes with a rather constant natural relative abundance: ^74^Se (0.89%), ^76^Se (9.37%), ^77^Se (7.63%), ^78^Se (23.77%), ^80^Se (49.61%), and ^82^Se (8.73%). Interestingly, each of these selenium isotopes can be enriched to >99% and isotopically labeled selenium compounds such as selenite, selenomethionine, or selenocysteine can readily be synthesized. When selenium is added as pure isotope, its incorporation into selenoproteins can be followed by ICP MS. The efficiency of selenium exchange in proteins is analyzed by size exclusion chromatography (SEC) coupled with ICP MS (SEC-ICP MS).

Here, we developed an innovative strategy to selectively label and trace selenoproteins with non-radioactive selenium-enriched isotopes (^76^Se, ^77^Se) in the HEK293 cell line. Cellular extracts were simultaneously analyzed by SEC-ICP MS and by western blots with distinct selenoprotein-specific antibodies. We performed dose–response and kinetic experiments to study the hierarchy of selenoproteome regulation in this particular cell line. Our work further illustrates the potential for non-radioactive selenium-labeling of selenoproteins in the wide field of proteomics.

## 2. Results

### 2.1. Fractionation of Intracellular Selenocompounds from HEK293 by Size-Exclusion Chromatography Followed by Inductively Coupled Plasma Mass Spectrometry Detection (SEC-ICP MS)

**Figure 1:**
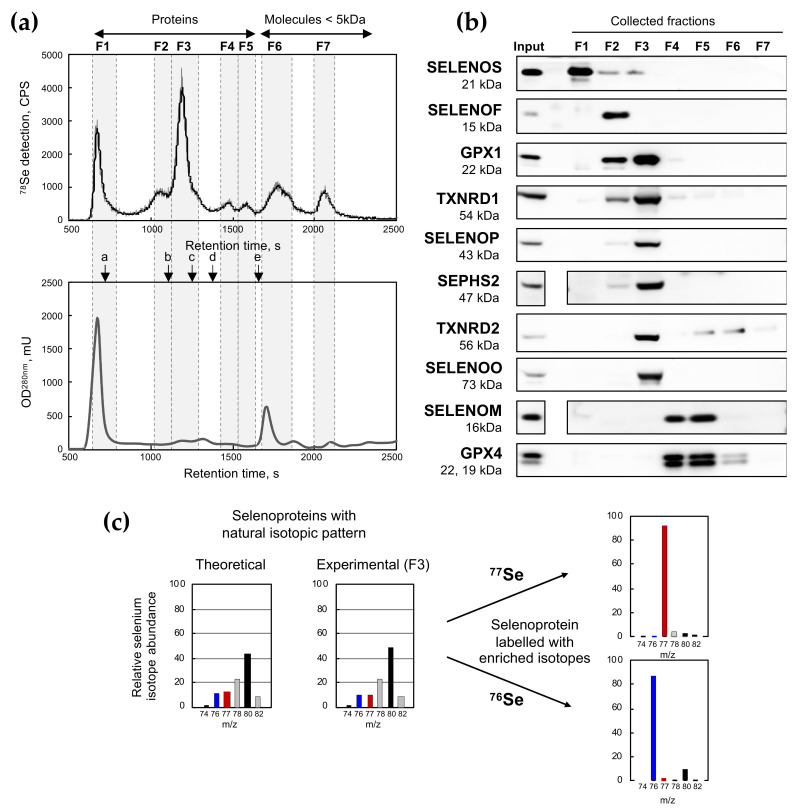
Fractionation of selenoproteins and selenocompounds of HEK293 cells by SEC-ICP MS. (**a**) Cellular extracts of HEK293 cells grown in Unsup + 100 nM of selenium (sodium selenite with natural isotopic pattern) were analyzed by SEC-ICP MS. The chromatographic profiles for ^78^Se and OD^280nm^ are represented in black and gray lines, respectively. The elution time of the selected calibration standards is shown in the bottom panel (a, thyroglobulin; b, transferrin; c, Mn-SOD; d, myoglobin; e, selenomethionine). The selenium eluted in seven fractions (referred to as F1 to F7) as noted from the ^78^Se chromatographic profile. Elution time and molecular weight range of these fractions are reported in Table 1. (**b**) The collected fractions F1 to F7 were then analyzed for the presence of 10 selenoproteins (SELENOS, SELENOF, GPX1, TXNRD1, SELENOP, SEPHS2, TXNRD2, SELENOO, SELENOM, and GPX4) using specific antibodies by western immunoblotting in comparison with the raw cellular extract. (**c**) Representation of selenium isotopic pattern in fraction F3 from fractionation SEC-ICP MS performed in panel (**a**). Cellular extracts of HEK293 grown in Unsup +100 nM of selenium (^76^Se or ^77^Se enriched selenite) were also analyzed by SEC-ICP MS. The isotopic enrichment of fraction F3 is illustrated in the two right panels.

The fractionation of HEK293 cellular extracts (500 µg of proteins) was reproducibly achieved in native conditions by size exclusion chromatography (SEC). The Superdex 200 used here allowed the separation of protein complexes in a range of molecular weights (MW) comprised between 10 kDa and 600 kDa, with heavier proteins or complexes eluting first (see Figure 1 and Table 1). In our experiment, SEC was linked to an UV^280nm^ monitor followed by an ICP MS tuned for a multi-isotopic selenium detection. Seven fractions were defined according to the selenium signal from SEC-ICP MS chromatograms (Figure 1 and Table 1). F1 to F5 were expected to contain selenoproteins while F6 and F7 should correspond to small MW selenocompounds such as selenite, mono- and di-methyl selenium, selenocyanate and potentially others. When cells were grown in a medium containing 100 nM of selenium, we found that selenoproteins were the major forms of selenocompounds found in cellular extracts. Indeed, the sum of the selenium contained in fractions F1 to F5 reached 75% of the total signal in the chromatogram (Figure 1a and Table 1).

The presence of 10 important proteins in the selenoproteome expressed in HEK293 cells was assayed in each fraction by western immunoblotting (Figure 1b). In elution fraction F1, corresponding to the exclusion volume (V_0_) of the column, we detected the presence of SELENOS, a rather small protein (21 kDa) that was previously found in a large complex from the endoplasmic reticulum (ER) [30]. Obviously, other selenoproteins other than those tested here may participate in the selenium signal detected in F1. In F2, SELENOF (also known as Sel15 or Sep15) which is also an ER-protein was detected. SELENOF (15 kDa) associates with the UDP-glucose:glycoprotein glucosyltransferase (UGTR) to form a 150–kDa complex [30]. GPX1 and TXNRDs (TXNRD1 and TXNRD2) that form tetramers (~80 kDa) and dimers (~110 kDa), respectively, were observed in F2 and F3. Other selenoproteins studied here (SELENOP, SEPHS2, SELENOO, SELENOM, and GPX4) are believed [6,8] to be predominantly in the monomeric form, and therefore eluted in the expected respective fractions (see Figure 1 and Table 1).

Interestingly, F3 contained at least six selenoproteins (GPX1, TXNRD1, SELENOP, SEPHS2, TXNRD2, and SELENOO) which accounted for almost 40% of the total selenium content of the extract (Table 1). We knew from previous work that the amount of selenium contained in GPX1 (present here in F3) represented approximately half of the total selenium in HEK293 selenoproteins when grown in a selenium-rich medium [26]. Consequently, F3 appeared as one of the most representative fractions for selenoproteome analyses. When the selenium isotopic pattern of F3 was analyzed, it fitted the theoretical selenium profile (Figure 1c). Then, when natural selenium selenite was replaced by either 100 nM of ^76^Se- and ^77^Se-enriched selenite in the culture media, the selenium isotopic profile of F3 was dramatically altered after three days of incubation as illustrated in Figure 1c. Notably, with both ^76^Se- and ^77^Se-enriched selenite, almost 90% of the selenoproteins in F3 contained the corresponding isotope, indicating an efficient and rapid exchange of the natural isotope by the enriched one. Our data confirm that isotopically enriched selenium can be used to efficiently label and trace selenoprotein expression in human cultured cell lines.

### 2.2. Selenium Assimilation in HEK293 Cells as a Function of Selenium Levels

**Figure 2:**
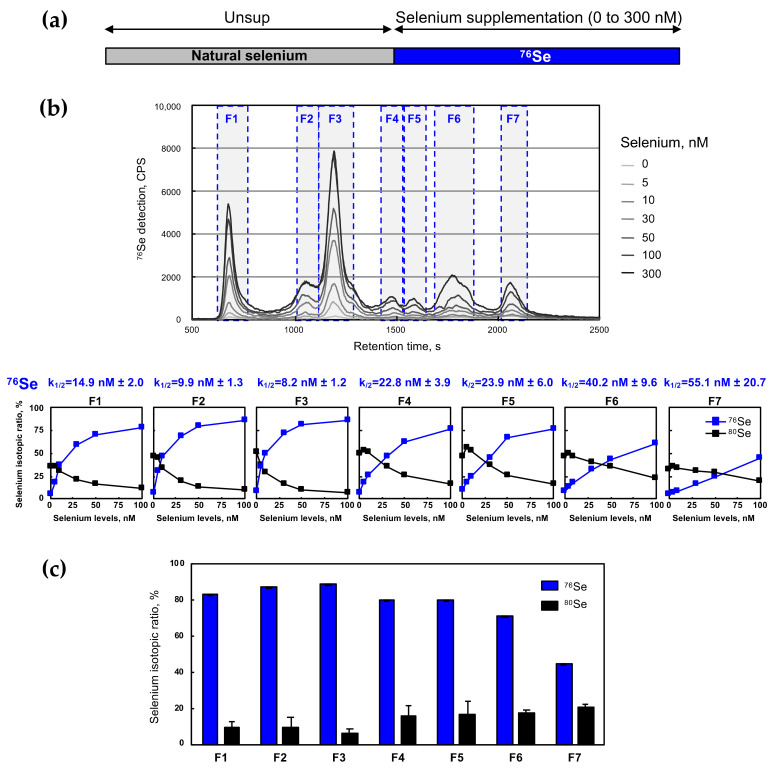
Analysis of HEK193 cells labeled with increasing concentrations of ^76^Se by SEC ICP MS. (**a**) Cells were grown for three days in a Dpl medium prior to being plated in fresh medium with increasing concentrations of ^76^Se enriched selenium (from 0 to 300 nM range). (**b**) The different cellular extracts were fractionated by SEC and all selenium isotopes (^74^Se, ^76^Se, ^77^Se, ^78^Se, ^80^Se and ^82^Se) followed by ICP MS detection. The SEC elution profile for ^76^Se is shown in the bottom panel for the various cellular extracts. The isotopic pattern was analyzed in the seven identified fractions, as indicated by dashed boxes (F1 to F7). The dose–response curves for ^76^Se abundance in each fraction as a function of selenium levels in the culture media were fitted as described in experimental procedures. The results for k_1/2_ for ^76^Se labeling are indicated on top of each graph. For clarity, only ^76^Se and ^80^Se are represented in blue and black squares, respectively, at a smaller scale of selenium levels (0 to 100 nM). (**c**) The selenium isotopic ratio is represented for the two most abundant isotopes (^76^Se and ^80^Se), for each fraction (F1 to F7), in the condition where 300 nM ^76^Se was used.

The selenium level in the growth media is known to modulate selenoprotein expression following a prioritized response, also called a hierarchy, but various selenium sources and concentrations have been used in the literature [31]. When 100 nM was added to the culture medium, the selenite induced a selective upregulation of the selenoproteins that was associated with an improved antioxidant defense [15]. Here, we aimed to define the optimal selenium levels (in the form of selenite) for each selenoprotein expression. To do so, increasing concentrations of ^76^Se-enriched selenite (from 5 to 300 nM) were added to the culture media after a preliminary selenium deprivation of the cells for three days, as illustrated in Figure 2a. In HEK293 cells, the toxic activity of selenite appears at concentrations higher than 1 µM [32]. At these higher levels, an adaptive response to selenium stress may occur. Cell extracts were harvested after three more days of incubation and analyzed by SEC-ICP MS, as mentioned above. The chromatograms (Figure 2b) showed that the ICP MS signal of ^76^Se increased in all fractions as selenium was added to the cell growth media. Interestingly, the highest selenium concentration used here (i.e., 300 nM) was not sufficient to reach a similar ^76^Se-isotope enrichment for all the selenocompounds (Figure 2c). Indeed, when a concentration of 300 nM of ^76^Se selenite was used, a ^76^Se-enrichment of 80–90% was observed for the protein fractions F1 to F5, while it reached 71% and 45% only in F6 and F7, respectively. This finding indicated a prioritized assimilation of selenium into the selenoprotein pool, with F3 being the most concentrated in terms of the newly synthesized selenoproteins. Then, the isotopic ratio in each fraction was calculated and plotted as a function of the added selenium. Only the most naturally abundant (^80^Se) and the supplemented isotope (^76^Se) are represented in Figure 2b. The curve-fit of the ^76^Se signal with a saturation equation gave us a half-saturation constant (k_1/2_) for each fraction (see Figure 2b). This value expressed the concentration of the added selenium which was necessary to exchange half of the natural selenium with the isotopically enriched element. The comparison of these different values provided the hierarchy of selenium assimilation into the different fractions. It appeared that selenium was primarily incorporated in the selenoprotein pool rather than in the metabolite fractions, with the following hierarchy: F2 and F3 > F1 > F4 and F5 > F6 and F7. Taken together, our data demonstrated that one cell passage of three days in selenium-supplemented conditions was sufficient to efficiently incorporate the isotopically enriched selenite in the newly synthesized selenoproteins.

### 2.3. The Selenoproteome Was Differentially Modulated According to the Selenium Level

**Figure 3:**
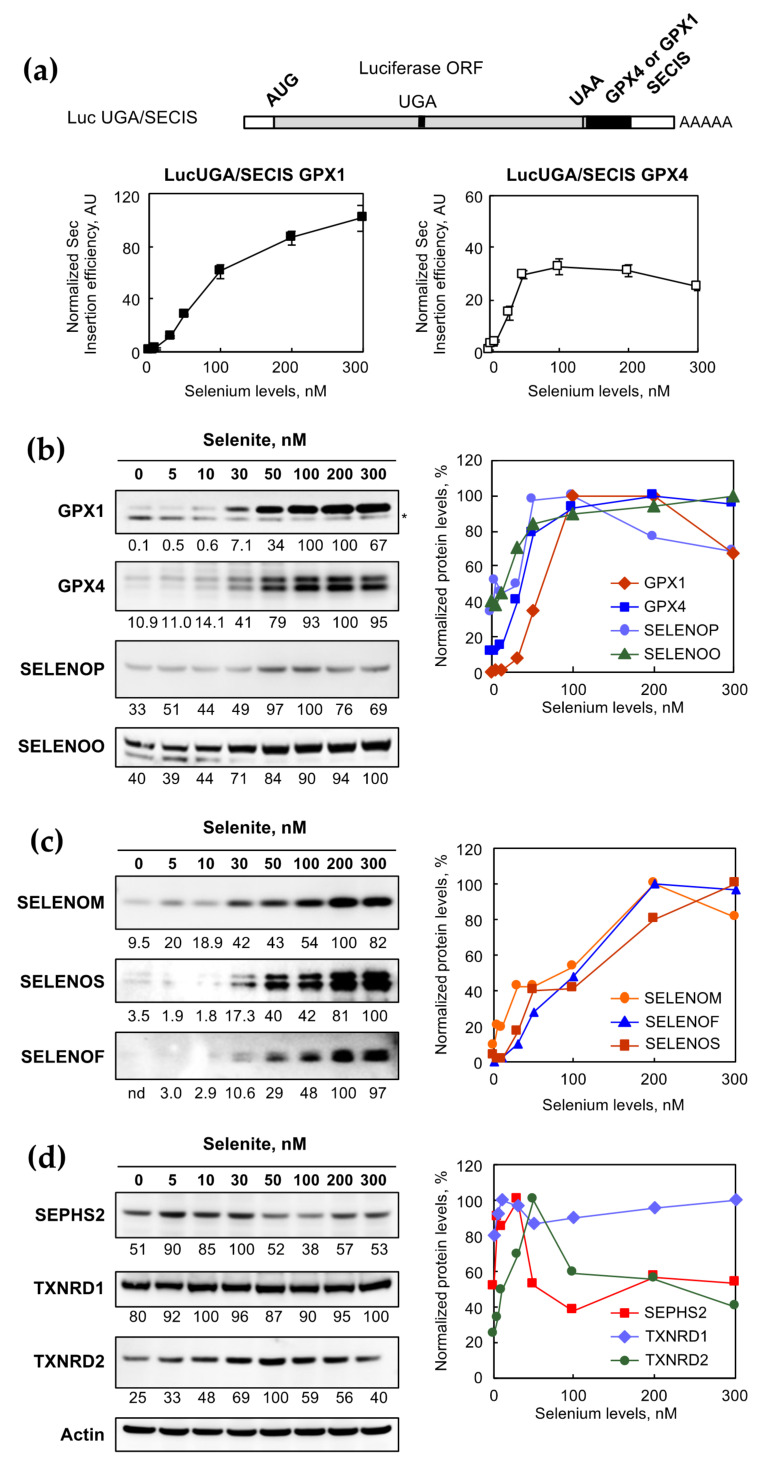
Analysis of selenoprotein expression as a function of selenium levels. (**a**) The response of selenocysteine insertion efficiency to selenium levels depends on the nature of the SECIS element. Two HEK293 cell lines stably expressing luciferase-based reporter constructs varying from the nature of the SECIS element, GPX1 and GPX4, respectively, were grown with increasing concentrations of selenium in the culture media. The structure of the Luc UGA/SECIS constructs was schematized. The Sec insertion efficiency was revealed by the luciferase activity normalized over the amount of proteins in cellular extracts. In both cell lines, the Sec efficiency is expressed relative to the one measured in the Dpl condition, set as 1. The HEK293 cell extracts with increasing amounts of ^76^Se selenite (similar to Figure 2) were also analyzed by western immunobotting for the presence of selenoproteins GPX1, GPX4, SELENOP, and SELENOO (**b**), SELENOM, SELENOS, and SELENOF (**c**), and SEPHS2, TXNRD1, and TXNRD2 (**d**). Relative quantification of individual selenoprotein over actin is indicated at the bottom of the corresponding immunoblot, with maximum intensity set at 100. The quantification results are also represented as a function of selenium levels in the culture media and sorted into three classes. The optimal selenoprotein expression is either obtained with a low-dose (top-right panel), high-dose (middle-right panel) or in a narrow range (bottom-right panel) of selenium concentration.

It has been established that selenoprotein expression is mostly controlled at the translational level in cultured cells in response to the changes in the selenium concentration. In HEK293 cells, selenoprotein mRNAs remained virtually insensitive to the selenium level [14,23]. To grasp this translational regulation, we have previously engineered and validated a set of luciferase-based reporter constructs [15,22,33]. HEK293 cells stably expressing Luc UGA/SECIS GPX1 or Luc UGA/SECIS GPX4 allowed the direct and rapid evaluation of Sec insertion efficiencies in response to various stimuli. Cells were grown with different selenium concentrations as indicated in Figure 3a. Then, cellular extracts were assayed for the luciferase activities, normalized to protein concentrations, and expressed relative to the activity measured in zero-selenium added conditions (unsupplemented, Unsup). Therefore, Figure 3a illustrates the fold stimulation of UGA recoding efficiency in response to the concentration of added selenite. Interestingly, we noticed an important dose–response increase in the Sec insertion with both constructs, with a maximum of a 100- and 50-fold stimulation, respectively, for Luc UGA/SECIS GPX1 and Luc UGA/SECIS GPX4. The Sec insertion efficiency reached a plateau at 50 nM selenium for the Luc UGA/SECIS GPX4 construct, while higher selenium levels seemed to be necessary for the Luc UGA/SECIS GPX1 constructs. These data further confirm the importance of translational control in the selenium-dependent modulation of selenoproteins.

To complement this analysis individually at the selenoprotein level, we performed western immunoblots with the cellular extracts from cells grown in the presence of various selenium levels (^76^Se). Ten selenoproteins that are well expressed and readily detectable with validated antibodies in HEK293 cells were assayed [14,23], and the protein contents in every condition were normalized respective to actin levels. These normalized selenoprotein levels were plotted as a function of the added selenium in the media. It appeared that three main categories of selenium responses emerged from our experiments, as shown in Figure 3b–d. The first category included GPX1, GPX4, SELENOP, and SELENOO, the levels of which were sensitive to selenium variation and reached an optimal expression level at a 100 nM selenium concentration or lower (Figure 3b). The second category comprised SELENOM, SELENOS, and SELENOF, which reached a maximum expression only at a 200 nM selenium concentration or higher (Figure 3c). The first and second categories were similar and fall into the “stress-related” family of selenoproteins. The last category included “house-keeping” selenoproteins, namely SEPHS2, TXNRD1, and TXNRD2, and had a very distinct response to selenium supplementation. They were either sensitive to the selenium variation with an inverse U-shaped curve (SEPHS2 and TXNRD2) or barely sensitive (TXNRD1), as shown in Figure 3d. Taken together, our data confirmed that the selenoproteome was highly modulated by selenium level variation in HEK293, with a specific hierarchy, mostly controlled at the translation stage.

### 2.4. Kinetics of Selenium Assimilation by HEK293 Cells as Selenoproteins and as Low-Molecular Selenocompounds

**Figure 4:**
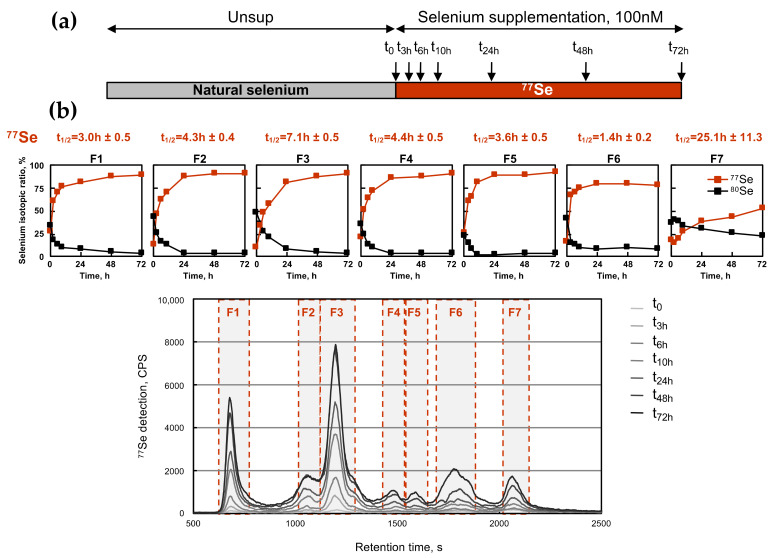
Time-course analysis of HEK193 cells labeled with 100 nM of ^77^Se by SEC ICP MS. (**a**) Cells were grown for three days in a Dpl medium prior to being plated in fresh medium with 100 nM of ^76^Se enriched selenium. Then, the cellular extracts were harvested at defined times post treatment (0, 3, 6, 10, 24, 48, and 72 h) and analyzed by SEC-ICP MS as described in Figure 2. (**b**) The chromatographic profile for ^77^Se is shown in the bottom panel for the various cellular extracts. The isotopic pattern was analyzed in the seven identified fractions, as indicated by dashed boxes (F1 to F7). Only ^77^Se and ^80^Se are represented in red and black squares, respectively. The kinetics for ^77^Se abundance in each fraction as a function of time were fitted as described in experimental procedures. The results of t_1/2_ for ^76^Se labeling are indicated on top of respective graphs.

Then, we used the isotopically enriched selenite to analyze the kinetics of the selenium incorporation in selenoproteins. In this experiment, cells were grown in conditions with a limited amount of selenium (Unsup condition) for 3 days and then passaged in a growth medium supplemented with 100 nM of ^77^Se-selenite, as illustrated in Figure 4a. The HEK293 cells were harvested at different time points (0, 3, 6, 10, 24, 48, and 72 h), and analyzed for the selenium isotopic composition by SEC-ICP MS (see Figure 4b). For each of the seven fractions producing a selenium signal, the isotopic ratio was calculated, and then plotted as a function of time (see the seven histograms above the chromatograms in Figure 4b). As mentioned before, only ^80^Se and ^77^Se were represented in these histograms. The ^77^Se signal was analyzed with an exponential curve-fit to determine the time in the individual fraction when the half replacement of the original isotope was reached, and this time was referred to as t_1/2_ (see in Figure 4a). The t_1/2_ value illustrated the kinetic hierarchy of selenium assimilation by the cell between each fraction, regardless of whether they contained selenoproteins or other selenocompounds. Our experiments indicated that among all the fractions, F6 was the one which assimilated the newly introduced ^77^Se isotope the fastest. The half replacement of the natural isotopes occurred after a 1.5 h selenium-supplementation. Then, selenoprotein fractions (F1 to F5) came second in the kinetic hierarchy with t_1/2_ between 3.0 and 7.1 h (see Figure 4b). Finally, the selenium metabolite fraction F7 was the one which was the least efficient and the least rapid to incorporate ^77^Se, with a t_1/2_ longer than one day. Obviously, the selenium exchange in fraction F7 was rather inefficient, even after one passage. Our data suggest that selenium was rapidly assimilated in the selenoprotein fraction, and that one passage was sufficient to reach the plateau. Even if SEC-ICP MS did not allow the differentiation between individual selenoproteins, it provided important information about the hierarchy between selenoproteins and other selenocompounds.

### 2.5. Selenocysteine Insertion in Selenoprotein Was Timely Controlled in Response to Selenium Supplementation

**Figure 5:**
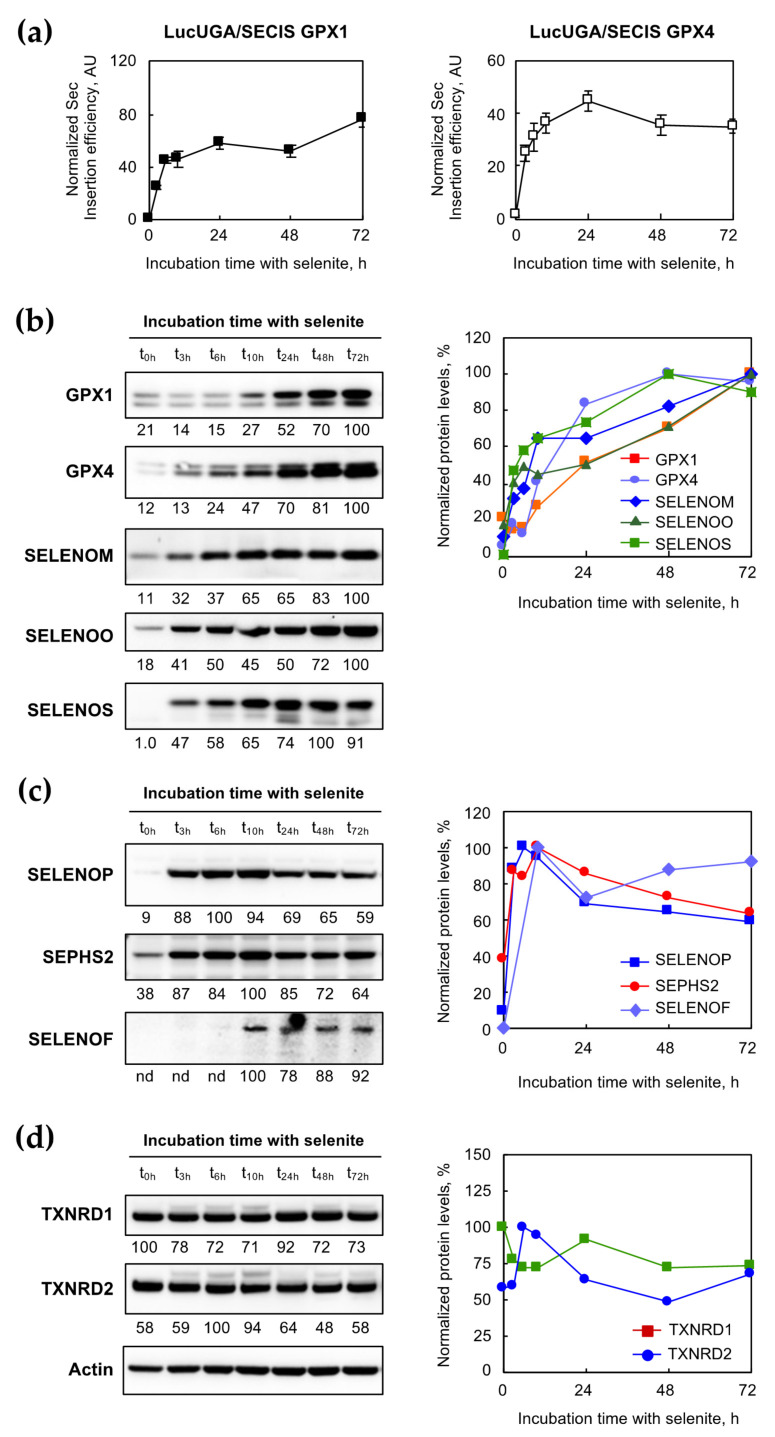
Time-course analysis of selenoprotein expression in medium containing 100 nM ^77^Se. (**a**) The stimulation of selenocysteine insertion in response to selenium addition is not dependent on the nature of the SECIS element. The two cell lines containing Luc UGA/GPX1 or Luc UGA/GPX4 construct were grown for three days in a Dpl medium prior to being plated in medium containing 100 nM of selenium and harvested at a defined time as described in Figure 4. Cell extracts were analyzed for luciferase activities and Sec efficiency was expressed relative to the one measured at t_0_, set as 1. The HEK293 cell extracts labeled with ^77^Se (similar to Figure 4) were analyzed by western immunobotting for the presence of selenoproteins GPX1, GPX4, SELENOM, SELENOO, and SELENOS (**b**), SELENOP, SEPHS2, and SELENOF (**c**), and TXNRD1, and TXNRD2 (**d**). Relative quantification of individual selenoprotein over actin is indicated at the bottom of the corresponding immunoblot, with maximum intensity set at 100. The quantification results are also represented as a function of time and sorted into three classes. The optimal selenoprotein expression was reached either after 48 h (top-right panel) or before 10 h (middle-right panel). Alternatively, the expression of selenoprotein can be stable with time (bottom-right panel).

We took advantage of our luciferase-stable expressing cell lines to further analyze how fast the translational UGA/Sec recoding machinery responded to selenium supplementation (cf. Figure 5a). Clearly, with both constructs, a rapid and massive increase in Sec insertion was observed at the first time point (3 h) to reach a plateau at approximately 6 h after a 100 nM selenium supplementation of the culture media. These data indicate that the translational machinery switched very quickly from an inefficient to an efficient state in response to selenium concentration variation.

In order to obtain information about the kinetic hierarchy between individual selenoproteins, we performed a western immunoblot with the extracts harvested at different time points. Again, three categories of selenoproteins can be deduced from the kinetic behavior, as shown in Figure 5b–d. The first category may refer to the so-called stress-related selenoproteins, with a rather slow increase in protein expression in response to the selenium supplementation. This category, composed of GPX1, GPX4, SELENOM, SELENOO, and SELENOS (Figure 5b), reached the maximum expression between 48 and 72 h. In contrast to that, the second category of selenoproteins, composed of SELENOP, SEPHS2, and SELENOF (Figure 5c), was characterized by a rapid and massive stimulation of protein expression, with maximal expression at 6 h for SELENOP or after 10 h for SELENOF and SEPHS2. Note that a decrease in the protein expression was observed at 72 h in the case of SELENOP and SEPHS2. This biphasic kinetic may reflect a negative feedback control of gene expression. The third category of selenoproteins, composed of TXNRD1 and TXNRD2 (Figure 5d), was poorly regulated over time by selenium supplementation and can be clearly associated with “housekeeping” selenoproteins. Taken together, our data indicated that the selenoproteome was highly regulated by selenium supplementation in HEK293, with specific individual kinetics.

### 2.6. Kinetics of Selenium Exchange in HEK293 Grown in Selenium-Supplemented Conditions

**Figure 6:**
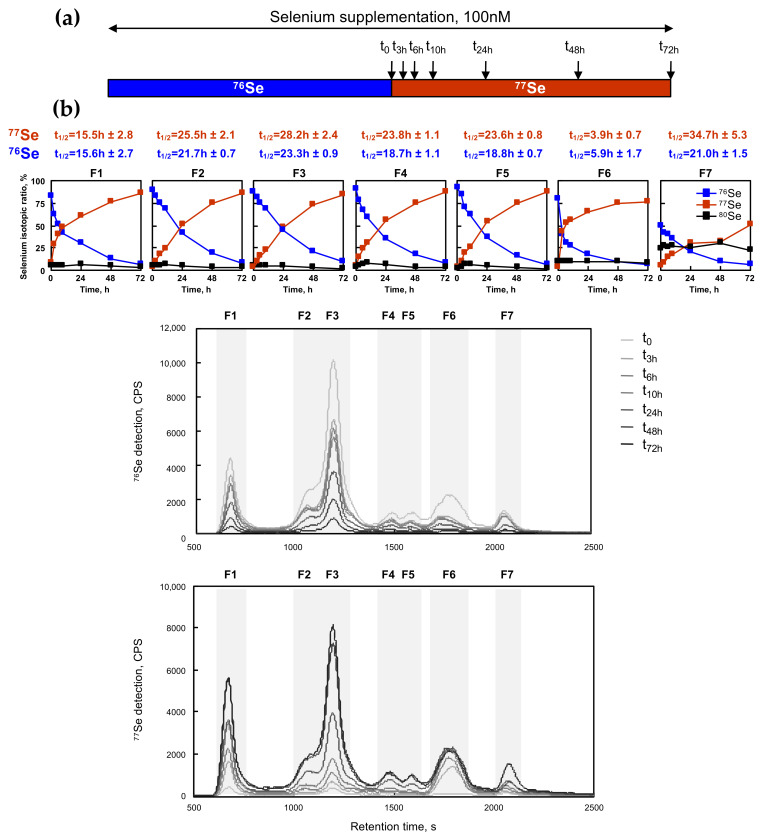
Time-course analysis of selenoprotein expression in medium containing 100 nM ^77^Se. (**a**) Cells were grown for three days in a medium containing ^76^Se (100 nM) prior to being plated in a fresh medium with 100 nM of ^77^Se enriched selenium. Then, the cellular extracts were harvested at defined time post treatment (0, 3, 6, 10, 24, 48, and 72 h) and analyzed by SEC-ICP MS as described in Figure 4. (**b**) The chromatographic profiles for ^76^Se and ^77^Se are shown for the various cellular extracts in the bottom two panels. The isotopic pattern was analyzed in the seven identified fractions, as indicated by dashed boxes (F1 to F7). ^76^Se, ^77^Se and ^80^Se are represented in blue, red and black squares, respectively. The kinetics for ^76^Se and ^77^Se abundance in each fraction as a function of time were fitted as described in experimental procedures. The results of t_1/2_ for ^76^Se and ^77^Se for each fraction are indicated on top of the respective graphs.

After having studied the response to selenium supplementation and having shown the ability to label the selenoproteome and selenocompounds with ^76^Se and ^77^Se enriched selenite, we then analyzed the half-lives of selenium in the different fractions detected by SEC-ICP MS, using constant conditions of selenium supplementation in the cell culture. To do so, we initially grew the HEK293 cells in 100 nM of ^76^Se selenite for three days and then passaged them into a fresh medium containing 100 nM of ^77^Se selenite, as illustrated in Figure 6. HEK293 cells were harvested at different time points (0, 3, 6, 10, 24, 48, and 72 h), and analyzed for the selenium isotopic composition by SEC-ICP MS as described earlier (see Figure 6b). The isotopic ratio was calculated in each fraction, and then plotted as a function of time. The signals of ^80^Se, ^76^Se, and ^77^Se were represented in the seven histograms above the chromatograms of Figure 6b. The signals of ^76^Se and ^77^Se were exponentially curve-fitted to extract t_1/2_, as described previously (see Figure 6a and Table 2). In almost every fraction, the deduced value of t_1/2_ was highly similar for the decrease in ^76^Se and the increased signal of ^77^Se, confirming the robustness of our method to label and trace the selenium signal in cultured cells. This t_1/2_ value indicated the rate by which selenium was exchanged in the molecules of each fraction, and was often referred to as the half-life. Indeed, at every time point, this value resulted from the balance between the neo-synthesis or intake (followed by the ^77^Se signal) and the degradation or secretion (followed by the ^76^Se signal). From our experiment, it appeared that F6 (metabolites) had the shortest half-life, below 6h. In contrast to that, fractions F2 to F5 containing selenoproteins had an average half-life of approximately one day (24 h). Interestingly, only F1 had a shorter t_1/2_ of 15 h, suggesting a variability within the selenoproteome. Again, a unique behavior was observed for fraction F7. The level of the initial ^80^Se stayed constant at 25% while ^76^Se and ^77^Se exchanged at t_1/2_ of 21 h and 35 h, respectively. These data suggest that only half of the selenium content in F7 was efficiently exchangeable, the other half is likely a long-term storage form of selenium. In summary, our data indicate that our strategy is efficient to label the selenoproteome with different isotopes and to trace the evolution within it. Note that the determination of the individual selenoproteins cannot be performed by western immunoblotting since antibodies are unable to differentiate between ^76^Se and ^77^Se.

## 3. Discussion

### 3.1. Hierarchy of Selenoproteins in Response to Selenium Supplementation: Kinetic and Dose–Response Analyses

Selenium is a vital component of selenoproteins and is co-translationally incorporated in the primary structure. As such and due to the fluctuations of its concentration in fluids in the body, there is a tight control of selenoprotein biogenesis by the selenium level. In this context of physiological selenium level variations, a hierarchical expression of the selenoproteome is established which maintains essential selenoproteins at the expense of the others. More importantly, among the stress-related selenoproteins, some are more sensitive than others to the changes in the selenium level and have different optimal expressions. This hierarchy of selenoprotein expression was described for the different glutathione peroxidases in rodents [34,35], but remain uncertain for other members. Yet, it becomes clear that most of this regulation occurs at the translational levels [11], but remains uncertain for other members. This classification between stress-related and housekeeping selenoproteins is not yet clear-cut but is also supported by unique features of Sec-tRNA^[Ser]Sec^ in mammals [36].

Our study shows that the optimal selenium level for a maximum selenoprotein expression varies from one protein to another. We validated this finding in HEK293 cells, and we anticipate that this could also stand true in other cell lines. We observed that several selenoproteins, including GPX1, GPX4, SELENOO, SELENOM, and SELENOS had a similar dose–response and kinetic behavior in response to selenium supplementation. These selenoproteins are among the most responsive to the selenium level variation in terms of time and concentration. On the other side, TXNRD1 and TXNRD2 are poorly sensitive to the selenium level variation and can be considered as housekeeping members. An interesting selenoprotein in terms of selenium regulation is SEPHS2, for which a transient overexpression occurs as a function of selenium concentration and also as a function of time after selenium supplementation. Our data clearly suggest a tight control of this protein level with potential negative feedback. A remarkable characteristic of SEPHS2 is that it is implicated in selenoprotein biogenesis by producing a Sec-tRNA^[Ser]Sec^ precursor, the selenophosphate (H_2_O_3_PSe^−^). This process is strictly dependent on the bioavailability of intracellular selenium. Thus, the fact that SEPHS2 also contains a Sec residue in its catalytic site is a way of controlling the level of mature Sec-tRNA^[Ser]Sec^ and, therefore, the selenoproteome. The amplification phase of SEPHS2 expression is easily explained by this mechanism. However, the mechanism for the negative feedback phase remains to be clarified, both temporally and at higher selenium levels.

### 3.2. Selenium-Enriched Isotope Labeling: An Alternative to Radioactive Labeling and a Novel Multiplexing Strategy for Selenoproteomic Analyses

In this work, we have developed an innovative strategy to label and trace selenoproteins in cultured cell lines, using isotopically enriched forms of selenium. In practice, six naturally present stable isotopes, namely ^74^Se, ^76^Se, ^77^Se, ^78^Se, ^80^Se, and ^82^Se can be used and are commercially available. Interestingly, the signal for these isotopes can be followed by either element (ICP ionization) or molecular (electrospray ionization) mass spectrometry. Here, we validated the potential of this strategy in HEK293 cells, but it can be applied to virtually any cell line which synthesizes selenoproteins. The use of SEC-ICP MS seems to be a robust and quick way to monitor the efficient labeling of a cellular extract. Our data demonstrate that selenite is readily and rapidly available for selenoprotein biogenesis. Our concept is likely to profit from recent advances in terms of proteomics, in terms of detection limits, concentration range, rapidity, and the number of proteins analyzed simultaneously [37]. All selenoproteins (except GPX6) have been detected from human samples in high throughput proteomics (www.proteomicsdb.org, accessed date 1 July 2021). The Sec-containing peptides resulting from tryptic digests have been detected and sequenced (by MS/MS fragmentation) for several human selenoproteins, including GPX1, GPX4, TXNRD1, TXNRD2, and SELENOF [26], but this could definitely be conducted for others. Additionally, the presence of selenium in a molecule such as a peptide can be inferred from its isotopic pattern. In this condition, the difference between protein abundance from one condition to another can be easily measured. Indeed, when using two selenium isotopes, pairs of selenopeptides that differ from selenium isotopes reflect the relative abundance of the corresponding protein, similar to what is conducted in stable isotope labeling with amino acids in cell culture (SILAC). The only, but important, difference with selenium-labeling versus SILAC is related to the availability of six selenium isotopes. Therefore, instead of looking at pairs of peptides, one could follow six different peptides simultaneously. This opens many multiplexing possibilities for selenoprotein labeling and tracing. One could consider that different cellular or tissue extracts originating from samples grown with various selenium isotopes could be quantified for selenoprotein contents using this multiplexing strategy. Non-radioactive multiple selenium-labeling of selenocompounds opens the way to quantitative selenoproteomics in the wide world of proteomics.

Recently, a selenocysteine-specific mass spectrometry-based technique was developed by the selective alkylation of selenocysteine [38]. This method allowed the systematic profiling and quantitative analysis of mouse selenoproteins, but can be applied to other species. In addition to known selenoprotein members described in [12], several novel candidates were proposed in which selenocysteine-containing peptides were characterized by mass spectrometry. In these peptides, the insertion of selenocysteine seemed to result from an inefficient UGA recoding event that occurs without a known SECIS element [38]. These data suggest that at least mammalian selenoproteomes could be more complex than previously expected. This new development in selenoproteome profiling can definitely benefit from selenium isotopic labeling and tracing that we have developed in our present study. 

## 4. Materials and Methods

This manuscript adopts the new systematic nomenclature of selenoprotein names [9].

### 4.1. Materials

The HEK293 cell line used in this study was purchased from Life technologies. For the HEK293 cells stably expressing Luc UGA/GPX1 or Luc UGA/GPX4, they were generated and validated in [15,22,33]. Fetal calf serum (FCS), cell culture media and supplements were purchased from ThermoFisher Scientific (Waltham, MA, USA). Transferrin, insulin, 3,5,3′-triiodothyronine, hydrocortisone, EDTA, sodium selenite, and DTT were purchased from Merck (Darmstadt, Germany). ^76^Se (99.8%) and ^77^Se (99.2%) isotopically enriched selenites were provided by Isoflex (San Francisco, CA, USA). Antibodies were purchased from Abcam (Cambridge, UK) (GPX1, #ab108429; GPX4, #ab125066; SELENOF, #ab124840; SELENOM, #ab133681; SELENOO, #ab172957; SELENOP, #ab109514), ThermoFisher Scientific (TXNRD1, #LF-MA0015) and Merck (SELENOS, #HPA010025; TXNRD2, #HPA003323; SEPHS2, #WH0022928M2; Actin, #A1978). NuPAGE 4–12% bis–Tris polyacrylamide gels, MOPS, MES SDS running buffers and antiprotease inhibitor cocktail were purchased from ThermoFisher Scientific.

### 4.2. Cell Culture and Incubation with Different Selenium Doses

HEK293 cells were grown and maintained in 75 or 150 cm^2^ plates in Dulbecco’s Modified Eagle Medium (D-MEM) supplemented with a 2% fetal calf serum (FCS), 100 μg mL^−1^ streptomycin, 100 UI mL^−1^ penicillin, 1 mM sodium pyruvate, 2 mM l-glutamine, 5 mg L^−1^ transferrin, 10 mg L^−1^ insulin, 100 pM 3,5,3′-triiodothyronine (T3), and 50 nM hydrocortisone. This medium is referred to as Unsup and contains 3 nM of selenium as determined in [15,16,22,33]. Cells were cultivated in 5% CO_2_ at 37 °C in a humidified atmosphere. The addition of selenium was performed with sodium selenite, either natural (Merck) or isotopically enriched (Isoflex), at the concentration indicated in figure legends for each experiment. After the treatment, cellular extracts were harvested in a 300 µL passive lysis buffer containing 25 mM of Tris phosphate at a pH of 7.8, 2 mM DTT, 2 mM EDTA, 1% Triton X100, and 10% glycerol. Then, protein concentrations were measured using the DC kit protein assay kit (Biorad, Hercules, CA, USA) in microplate assays using the microplate reader FLUOstar OPTIMA (BMG Labtech, Champigny-sur-Marne, France).

### 4.3. Evaluation of Selenocysteine Insertion Efficiency

To analyze Sec insertion efficiency in HEK293, we used luciferase-based reporter constructs that were validated for UGA/Sec recoding in transfected cells [22,33]. Briefly, the minimal SECIS elements from GPX1 and GPX4 were cloned downstream of a luciferase coding sequence, which was modified to contain an in frame UGA codon at position 258 (Luc UGA/SECIS), as shown in Figure 3a. HEK293 cells stably expressing Luc UGA/GPX1 and Luc UGA/GPX1 SECIS were previously generated and validated [15,22]. After being grown in various concentrations of sodium selenite, cells were harvested and the cellular extracts were assayed for luciferase activities by chemiluminescence (Luciferase assay systems, Promega, Charbonnières, France), in triplicate using a microplate reader FLUOstar OPTIMA (BMG Labtech). We arbitrarily expressed the Sec insertion efficiency relative to the luciferase activity measured in Unsup conditions, which was set at 1.

### 4.4. Fractionation of Selenium Containing Molecules by Size Exclusion Chromatography (SEC) with ICP MS Detection (SEC-ICP MS)

A precise amount of cellular extract, corresponding to 500 µg of proteins, was fractionated by HPLC (Agilent 1200 series, Santa Clara, CA, USA) using a SEC column (Superdex 200 10/300 GL, GE Healthcare, Chicago, IL, USA). The flow rate was set at 0.7 mL min^−1^ of a mobile phase (ammonium acetate buffered at pH 7.4). The injection volume was 100 μL. The calibration of the SEC column was performed using protein standards (thyroglobulin 670 kDa, transferrin 81 kDa, bovine albumin 66 kDa, chicken albumin 44 kDa, Mn-SOD 39.5 kDa, Cu/Zn SOD 32.5 kDa, carbonate dehydratase 29 kDa, myoglobin 16 kDa, metallothionein 6.8 kDa, and selenomethionine 0.198 kDa). Detection was achieved online by recording the absorbance at 280 nm (Agilent G1365B) and by ICP MS (Agilent 7500) for multi-isotopic detection (^74^Se, ^76^Se, ^77^Se, ^78^Se, ^80^Se, and ^82^Se) with an integration time per element of 0.1 s. UV^280nm^ and ICP MS signals were exported and treated with Microsoft Excel software to present chromatograms and isotopic graphs. Seven chromatographic fractions were defined with the elution times listed in Table 1. To integrate the total count of each selenium isotope ion per fraction, the background levels were determined and subtracted. The dose–response and kinetic curves were fitted using Kaleidragraph software with standard equations. For dose–response curves, it was as follows: y = a + ((b.x)/(k + x)), where k was the concentration of selenium allowing half saturation of the signal, and therefore referred to as k_1/2_ throughout the manuscript. Kinetic curves were fitted with y = a + b.exp(−k.t), where the constant t_1/2_ = (ln(2)/k), and the represented time allowing an increase or decrease in the signal by 50%.

### 4.5. Protein Gels and Western Immunoblotting

Equal protein amounts (50 µg) were separated in Bis-Tris NuPAGE Novex Midi Gels and transferred onto nitrocellulose membranes using iBlot^®^ DRy blotting System (ThermoFisher Scientific). Membranes were probed with primary antibodies (as indicated) and HRP-conjugated anti-rabbit or anti-mouse secondary antibodies (Merck). The chemiluminescence signal was detected using the ECL Select Western Blotting Detection Kit (GE Healthcare) and the PXi 4 CCD camera (Ozyme, Saint-Cyr-l’École, France). Image acquisition and data quantifications were performed with the Syngene softwares, GeneSys and Genetools (Ozyme), respectively.

### 4.6. Large Scale Fraction Collection for Western Immunoblotting Analyses

Larger amounts of cellular extracts, corresponding to 5 mg of proteins, were loaded on an SEC column in the same configuration as before, but fractions were collected as indicated in Table 1 instead of going to an ICP MS detector. The seven different fractions were lyophilized and resuspended in 200 µL of the lysis buffer. An aliquot of 10 µL of each fraction was analyzed by western immunoblotting after migration onto protein gels.

## 5. Conclusions

The selenoproteome is expressed from 25 selenoprotein genes in humans, where selenocysteine incorporation is genetically encoded. Their expression is finely controlled by the selenium status of the organism or the availability of selenium in the culture medium. Here, we have developed a new analytical strategy to study the expression of the selenoproteome in response to selenium supplementation using non-radioactive selenium isotopes (^76^Se, ^77^Se). We characterized the selective regulation of the most abundant selenoproteins in dose–response and kinetic experiments in HEK293 cell line. Our data suggest that the use of other natural isotopes of selenium (^74^Se, ^78^Se, ^80^Se and ^82^Se) in multiplexing experiments followed by inductively coupled plasma mass spectrometry detection is entirely feasible. Non-radioactive labeling of cellular selenocompounds paves the way for quantitative selenoproteomics and selenometabolomics in the ever-improving world of elemental and molecular mass spectrometry.

## Figures and Tables

**Table 1:** Chromatographic data for each fraction collected according to the SEC-ICP MS chromatogram. n.d., not detected.

Fraction N°	Retention Time, s		MW Range, kDa	Selenium Signal Repartition, %						Selenoproteins Detected by Westernblot (Figure 1)
				Dpl			Dpl + 100 nM Se			
	Start	End		Ave		SD	Ave		SD	
F1	600	760	>480	12.0	±	0.6	17.5	±	0.9	SelenoS
F2	1000	1120	140–75	4.7	±	0.2	10.2	±	0.5	SelenoF, Gpx1, TR1
F3	1120	1300	75–30	25.4	±	1.3	37.3	±	1.9	Gpx1, Txnrd1, SelenoP, Sephs2, Txnrd2, SelenoO
F4	1410	1540	8–16	3.5	±	0.2	5.8	±	0,3	SelenoM, Gpx4
F5	1540	1650	4–8	2.7	±	0.1	4.4	±	0.2	SelenoM, Gpx4, Txnrd2
F6	1660	1870	1.5–4	24.7	±	1.2	16.5	±	0.8	Gpx4, Txnrd2
F7	1990	2130	0.38–0.80	27.1	±	1.4	8.4	±	0.4	n.d.

**Table 2:** Summary of the distinct features for each fraction collected according to the SEC-ICP MS chromatogram.

Fraction N°	Dose–Response (Figure 2)	Kinetics of Selenium-Labeling (Figure 4)	Kinetics of Selenium Exchange (Figure 6)	
	k_1/2_^76^Se, nM	t_1/2_^77^Se, h	t_1/2_^77^Se, h	t_1/2_^76^Se, h
F1	14.9 ± 2.0	3.0 ± 0.5	15.5 ± 2.8	15.6 ± 2.7
F2	9.9 ± 1.3	4.3 ± 0.4	25.5 ± 2.1	21.7 ± 0.7
F3	8.2 ± 1.2	7.1 ± 0.5	28.2 ± 2.4	23.3 ± 0.9
F4	22.8 ± 3.9	4.4 ± 0.5	23.8 ± 1.1	18.7 ± 1.1
F5	23.9 ± 6.0	3.6 ± 0.5	23.6 ± 0.8	18.8 ± 0.7
F6	40.2 ± 9.6	1.4 ± 0.2	3.9 ± 0.7	5.9 ± 1.7
F7	55.1 ± 20.7	25.1 ± 11.3	34.7 ± 5.3	21.0 ± 1.5

## Data Availability

Requests for further information about resources, reagents, and data availability should be directed to the corresponding author.
